# Comparison of Echocardiography and Invasive Transseptal Catheterization for Assessing Transvalvular Gradient in Patients with Surgical Aortic Valve Prostheses: Fact or Myth?

**DOI:** 10.3390/diagnostics15232980

**Published:** 2025-11-24

**Authors:** Ahmet Hakan Ates, Ahmet Kivrak, Ugur Canpolat, Mert Dogan, Gul Sinem Kılıc, Can Menemencioglu, Ugur Nadir Karakulak, Ergun Barıs Kaya, Mehmet Levent Sahiner, Kudret Aytemir

**Affiliations:** Department of Cardiology, Hacettepe University Faculty of Medicine, Ankara 06230, Turkey; ahmethakanates@yahoo.com (A.H.A.); drmertd@gmail.com (M.D.); kilicgulsinem@gmail.com (G.S.K.); c.menemencioglu@gmail.com (C.M.); ukarakulak@gmail.com (U.N.K.); doctorkaya@yahoo.com (E.B.K.); mleventsahiner@yahoo.com (M.L.S.); aytemirk@gmail.com (K.A.)

**Keywords:** aortic valvular prosthesis, transvalvular gradient, echocardiography, transseptal catheterization

## Abstract

**Background:** Echocardiography is the primary assessment tool for follow-up in patients with aortic valve prostheses. However, there are concerns regarding the consistency between echocardiographic and invasive transvalvular gradients (TVGs). This study utilized both noninvasive and invasive methods to compare the TVGs in aortic valve prostheses. **Methods:** The study included fourteen patients who had previously undergone surgical aortic valve replacement [metallic (*n* = 12) and bioprosthetic (*n* = 2)]. All patients had moderate-to-severe TVGs, which were measured during follow-up echocardiography, and they underwent invasive transseptal catheterization. Invasive and echocardiographic TVGs were measured and compared. **Results:** The median interval between index valvular surgery and invasive TVG measurement was 6.7 (2.5–11.5) years. The median interval between echocardiographic and invasive TVG measurements was 7.2 (2–19) days. Only 12 (85.7%) patients were symptomatic during echocardiographic assessment. Maximum TVGs obtained by echocardiography were higher than invasive peak-to-peak TVGs (77.0 ± 13.1 vs. 47.5 ± 21.7 mmHg, *p* < 0.05). There was a significant negative correlation between the echocardiography-based aortic valve area and the effective orifice area index with the catheter-based peak-to-peak aortic gradient (r = −0.64, *p* = 0.014 and r = −0.63, *p* = 0.015). Six patients (42.9%) who revealed severe catheter-based peak-to-peak aortic gradient underwent redo aortic valve surgeries. The cut-off value of EOAI of <0.50 cm^2^/m^2^ was found to be a predictor of severe catheter-based peak-to-peak aortic gradient. **Conclusions:** In our preliminary cohort study, the TVGs of aortic valvular prostheses measured by echocardiography were significantly greater than those measured by invasive transseptal catheterization. During follow-up, invasive confirmation of echocardiographic moderate-to-severe TVGs in selected patients with surgical aortic valvular prostheses may be considered.

## 1. Introduction

Aortic valve replacement (AVR), whether surgical or transcatheter, serves as the primary therapeutic option for symptomatic severe aortic stenosis (AS) or regurgitation (AR). It is vital to conduct structural and functional assessments of the replacement valve in these patients. Transthoracic echocardiography is a key tool for evaluating valvular function and structural integrity, primarily because of its noninvasive nature, wide availability, and absence of ionizing radiation exposure. Various echocardiographic parameters, including mean and peak transvalvular gradients (TVG), the dimensionless index (the ratio of left ventricular outflow tract (LVOT) to aortic valve time velocity integrals (TVIs)), effective orifice area (EOA) calculated using the continuity equation, and geometric orifice area determined by planimetry, can be used to assess valvular function. Nevertheless, numerous studies have highlighted significant discrepancies between echocardiographic and cardiac catheterization measurements, often referred to as “discordance.” This may be due to various factors, including but not limited to flow jet eccentricity, the pressure recovery phenomenon, limitations of the Bernoulli equation, flow rates, and prosthetic valve design [1,2,3].

Another critical factor influencing TVG is prosthesis-patient mismatch (PPM), which occurs when the EOA of a normally functioning prosthetic valve is too small in relation to the patient’s body size [4]. In surgically implanted aortic prostheses, PPM can result in elevated TVG, increased left ventricular afterload, and potential adverse outcomes. Its presence may also complicate echocardiographic assessments, making it challenging to differentiate true structural valve deterioration from flow-related TVG elevation [5]. Elevated TVG often indicates biomechanical stress and valve deterioration, making their reliability essential for clinical decision-making. Due to the limitations of echocardiography, alternative imaging methods or invasive assessments may be required to confirm elevated echocardiographic TVGs [6,7].

This study assessed whether elevated TVG identified during routine echocardiographic follow-up in patients with surgically implanted aortic valve prostheses reflected actual elevations by comparing these findings with invasive transseptal catheterization measurements.

## 2. Methods

### 2.1. Study Population

This retrospective cohort study included 14 patients with a history of surgical AVR between August 2018 and November 2024 [metallic (*n* = 12) and bioprosthetic (*n* = 2)]. Patients with increased mean and/or peak aortic TVG on routine echocardiographic follow-up or presented with symptoms suggestive of prosthetic valve dysfunction underwent invasive gradient measurements. Baseline demographic and clinical data were retrieved from patient files and the hospital’s electronic database. The study was conducted in accordance with the Declaration of Helsinki and was approved by the Hacettepe University Health Sciences Research Ethics Committee (Approval Date: 4 March 2025, Approval Number: 2025/06-19).

### 2.2. Echocardiographic Assessment

All patients underwent a comprehensive transthoracic echocardiographic evaluation using a Vivid E9 ultrasound system (manufactured by GE Vingmed Ultrasound, Horten, Norway). The same cardiologist analyzed all echocardiographic measurements without being aware of the patient’s clinical status. Standard 2D-based images and Doppler measurements, including parasternal and apical views, were obtained following the current expert consensus document [8,9].

**Figure 1:**
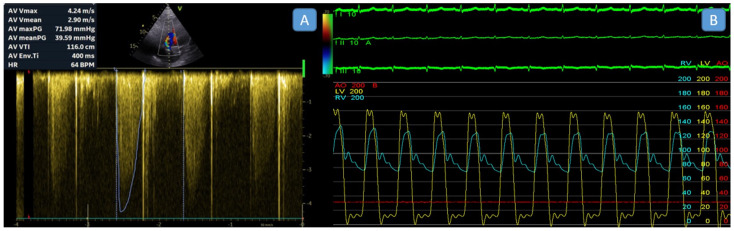
(**A**). Measurement of the gradient across the aortic mechanical valve by transthoracic echocardiography. (**B**). Simultaneous invasive measurement of aortic and left ventricular pressure gradients. The yellow line represents left ventricular pressure, and the blue line represents aortic pressure. In this patient, who had a surgical aortic valve replacement, transthoracic echocardiography indicated a pressure gradient of 59/38 mmHg across the aortic valve, while the invasive peak-to-peak gradient measurement was 19 mmHg.

The peak velocity, mean gradient, velocity time integral (VTI), Doppler velocity index (DVI), and effective orifice area (EOA) of the aortic prosthetic valve were measured and recorded. The simplified Bernoulli equation was employed to calculate pressure gradients across aortic prosthetic valves noninvasively. Peak aortic velocity and mean aortic gradients were derived from the flow envelope, using the highest velocity recorded from multiple imaging views. The mean gradient was obtained by tracing the Doppler envelope (Figure 1A).

The EOA of an aortic prosthesis was calculated as follows:

EOA = stroke volume/VTI_prosthetic valve_

where the VTI_prosthetic valve_ is the velocity-time integral through the prosthesis determined by CW Doppler. Stroke volume was derived as the cross-sectional area just proximal to the prosthesis multiplied by the VTI of flow by PW Doppler at that site.

In cases where the replacement valve is functioning normally but is too small for the size of the recipient, patient–prosthesis mismatch in the aortic position is defined by an EOA indexed to body surface area (EOAI) of less than 0.85 cm^2^/m^2^ and severe mismatch is indicated by an EOAI of less than 0.65 cm^2^/m^2^, with normal valvular appearance and normal echocardiographic findings parameters.

### 2.3. Invasive Transseptal Left Heart Catheterization

For patients on warfarin, transseptal puncture was performed after careful INR adjustment without LMWH bridging (INR 2.0–2.5). For patients on DOACs, the procedure was performed after 24 h interruption of the drugs without bridging (minimally interrupted anticoagulation approach without LMWH bridging).

The procedures were performed under conscious or deep sedation, ensuring patient comfort and procedural safety. For all patients, invasive hemodynamic measurements were obtained using a dual-catheter technique that allows for simultaneous recording of left ventricular (LV) and aortic pressures to assess TVG accurately. Two venous sheaths were placed in the femoral vein, and one arterial sheath was inserted into the femoral artery. A pigtail catheter was then positioned in the aortic root via the arterial sheath, and a diagnostic coronary sinus (CS) catheter was advanced into the CS through one of the venous sheaths to serve as markers for transseptal puncture. Using these markers as guidance, a transseptal puncture was performed employing the modified Brockenbrough technique under fluoroscopy visualization. After a successful puncture, a pigtail catheter was advanced into the left atrium and then across the mitral valve into the left ventricle (LV) (Video S1). This setup allowed for the simultaneous recording of left ventricular and aortic pressures, enabling accurate calculation of “peak-to-peak” transvalvular pressure gradients (Figure 1B). During the procedure, fluoroscopy was used to guide catheter placement and evaluate the motion and function of the prosthetic aortic valve leaflets, ensuring their proper operation during hemodynamic assessment. The dual-catheter approach is especially beneficial for patients with mechanical aortic valves, as it avoids crossing the valve apparatus, reducing the complication risk. Numerous studies have validated this method for its accuracy and reliability in measuring transvalvular pressure gradients, establishing it as a standard practice in invasive hemodynamic assessments [10,11].

### 2.4. Statistical Analysis

Statistical analyses were conducted using SPSS software (version 30.0). Descriptive variables were summarized as counts and percentages for categorical data. The normality of continuous variables was assessed using the Shapiro–Wilk test. Continuous variables following a normal distribution were expressed as mean ± standard deviation, while non-normally distributed data were presented as median and interquartile range. Group comparisons were performed using the independent Student’s *t*-test for normally distributed variables and the Mann–Whitney U test for non-normally distributed variables as appropriate. A two-tailed *p*-value of <0.05 was considered statistically significant.

## 3. Results

The baseline demographic and clinical characteristics of the 14 patients included in the study are outlined in Table 1, and a detailed comparison of echocardiographic and catheter-derived gradients is provided in Table 2. The mean age of the patients was 49.2 ± 16 years (range: 24–75 years), with the majority (71.4%) being female. Before an index valve surgery, all patients had primary severe AS, and one had an additional moderate AR. Among the cohort, 12 patients (85.7%) had mechanical bileaflet aortic valve prostheses, while the remaining two patients (14.3%) had bioprosthetic valves. Furthermore, 19 mm size was implanted in 3 patients (21.4%) and 21 mm size was preferred in the remaining 11 patients (78.6%). Although 9 patients (64.3%) were symptomatic on admission, the remaining 5 patients (35.7%) were asymptomatic. The median interval between aortic valve surgery and cardiac catheterization was 6.7 years, and between echocardiographic examination and cardiac catheterization was 7.2 days. The mean LVEF was 61.5 ± 4.5%, and the mean ascending aortic diameter was 33.4 ± 3.4 mm. Catheter-based measurements revealed a mean peak-to-peak transvalvular pressure gradient of 47.5 ± 21.74 mmHg, while the Doppler-derived peak gradient (77.0 ± 13.1 mmHg) was significantly higher (*p* < 0.05). Doppler-derived mean aortic gradient was 44.2 ± 8.9 mmHg. Correlation analysis revealed no correlation between echocardiography-based mean or peak aortic gradient and catheter-based peak-to-peak aortic gradient (*p* = 0.111, *p* = 0.705). However, there was a significant negative correlation between the echocardiography-based aortic valve area and catheter-based peak-to-peak aortic gradient (r = −0.64, *p* = 0.014). The EOAI also showed a significant negative correlation with catheter-based peak-to-peak aortic gradient (r = −0.63, *p* = 0.015). Six patients (42,9%) who revealed severe catheter-based peak-to-peak aortic gradient underwent redo aortic valve surgeries. The cut-off value of EOAI of <0.50 cm^2^/m^2^ was found to be a predictor of severe catheter-based peak-to-peak aortic gradient. A subgroup analysis was performed between mechanical (*n* = 12) and bioprosthetic (*n* = 2) valves; trends were similar but statistical power was limited.

## 4. Discussion

There are several scenarios regarding replacement valves in clinical practice, including normal functioning, obstructed due to pannus formation, thrombosis or vegetation, and PPM. In our study, we hypothesized a fourth scenario regarding the high-pressure gradient across aortic replacement valves, which could be defined as a discrepancy between echocardiography and catheter-based measurements caused by several factors.

Long-term follow-up of prosthetic valve function requires an accurate evaluation of valve hemodynamics, as it plays a crucial role in predicting the need for future interventions. While Doppler echocardiography is well-established in assessing native valve functions, its accuracy in determining prosthetic valve function remains controversial. Numerous studies have explored the concordance between transthoracic echocardiography and invasive catheter-based TVG measurements across various patient groups. Early research demonstrated strong correlations between these modalities in cases of native aortic valve stenosis [12]. However, subsequent investigations into prosthetic valves, including SAVR, TAVR, and ViV-TAVR, revealed notable discrepancies. While small-scale studies have provided limited data on Doppler–catheter concordance in prosthetic valve [13], more recent research consistently reports significant differences, with Doppler echocardiography frequently overestimating TVG. Our findings align with these previous observations, further emphasizing the limitations of Doppler methods in this context. A large multicenter study by Abbas et al. involving 808 TAVR patients demonstrated this phenomenon clearly: while good correlation existed between echocardiographic and invasive gradients in native aortic stenosis (r = 0.614), this correlation became weak to absent post-TAVR (r = 0.138), regardless of valve type or size [10]. Similarly, DeSa et al. demonstrated that only 26 patients (7.2%) had abnormal Doppler gradients immediately post-TAVR, but this increased to 109 patients (30.1%) at discharge, emphasizing the temporal evolution of this discordance phenomenon [14].

The discrepancy between echocardiographic and invasive measurements is multifactorial. Firstly, the time sequences of pressures in LVOT and aortic valves are not simultaneous. While the gradient between LVOT and aortic valve would be the “peak instantaneous gradient” in Doppler measurement, that would be the “peak to peak gradient” during invasive measurement. The latter is a more accurate value reflecting the pressure gradient across replacement valves. Secondly, the “pressure recovery phenomenon” may result in differences in peak instantaneous pressure readings across the aortic valve when measured using continuous wave-Doppler echocardiography and cardiac catheterization. In this phenomenon, kinetic energy gained across a narrowed orifice is partially converted back into pressure energy downstream. This phenomenon is particularly pronounced in patients with smaller aortic root diameters, leading to an underestimation of catheter-based gradients compared to Doppler-derived values [15]. Thirdly, Doppler-based methods have inherent technical limitations, including the assumptions of the simplified Bernoulli equation, which may not fully account for flow dynamics in prosthetic valves [16]. The Continuous-wave Doppler technique measures the maximal instantaneous blood flow velocity along the LVOT-aortic valve axis. This velocity is then converted into an estimated pressure gradient using the modified Bernoulli equation [6].

In contrast, catheterization directly measures the transvalvular pressure gradient (ΔP) distal to the aortic valve after pressure recovery has occurred between the left ventricle and the aorta. Additionally, Doppler echocardiography relies heavily on operator expertise, image quality, and accurate LVOT diameter measurements, while hemodynamic changes can influence catheter-based measurements during sedation or anesthesia [8]. The design of replacement valves, especially bileaflet mechanical valves, may contribute to incorrect Doppler-derived gradients. Studies have shown that bileaflet valves are particularly susceptible to localized high velocities and significant pressure recovery, which can lead to an underestimation of effective orifice areas and exaggerated Doppler gradients [17]. However, another in vitro study suggested that the Doppler–catheter gradient relationship might be independent of specific leaflet designs [1]. Furthermore, the degree of discordance may be influenced by the mechanism of valve dysfunction and the severity of stenosis, with evidence suggesting that this discordance increases as prosthetic stenosis becomes less severe [1]. Last but not least, suboptimal catheterization techniques, such as relying on catheter pullback instead of simultaneous left ventricular and aortic pressure measurements or using estimated Fick-derived cardiac output instead of direct measurement, may also contribute to the observed discrepancies [18]. Additionally, most catheterization measurements are performed under general anesthesia or conscious sedation, which can induce significant hemodynamic changes. These include reduced systemic vascular resistance, diminished myocardial contractility and stroke volume, and decreased oxygen demand, all of which can impact transvalvular pressure gradient measurements [19].

Another well-recognized phenomenon contributing to elevated Doppler-derived gradients is prosthesis–patient mismatch (PPM), which occurs when a normally functioning prosthetic valve is disproportionately small relative to the patient’s body size. This results in abnormally high transvalvular gradients and reduced EOA, ultimately increasing left ventricular afterload and affecting long-term outcomes [7,20]. The assessment of PPM, however, is often complicated by technical limitations, flow dependency, and variability in EOA measurement [21]. Herrmann et al. recently emphasized that the diagnostic accuracy of the indexed EOA (EOAi) may be compromised by measurement errors—particularly LVOT underestimation—and low-flow states, leading to “pseudo-PPM” and discordant findings between Doppler and invasive modalities [3]. In contrast to these concerns, our findings suggest that EOAi may, in fact, serve as a reliable surrogate for true hemodynamic obstruction in the setting of suspected prosthetic valve dysfunction. Notably, all patients in our cohort with an EOAi <0.50 cm^2^/m^2^ exhibited significantly elevated transvalvular gradients confirmed by transseptal catheterization. Furthermore, we observed a strong inverse correlation between EOAi and peak-to-peak invasive gradients (r = −0.63, *p* = 0.015). These results support the clinical utility of EOAi in stratifying patients for further invasive testing and suggest that a lower EOAi threshold (e.g., <0.50 cm^2^/m^2^) may be more predictive of clinically significant obstruction than conventional PPM cutoffs. Accordingly, in patients with high Doppler-derived gradients and low EOAi, early invasive assessment may be considered to guide appropriate management.

Periprocedural anticoagulation is a critical determinant of safety in procedures requiring left atrial access. Consistent with current evidence from AF ablation practice, uninterrupted vitamin K antagonist therapy is preferred over interruption and heparin bridging, as this strategy significantly reduces thromboembolic complications without increasing major bleeding risk [22]. In our cohort, patients receiving warfarin underwent transseptal puncture under uninterrupted anticoagulation with careful INR optimization (target INR 2.0–2.5), and DOAC-treated patients followed a minimally interrupted protocol without LMWH bridging. This approach mirrors contemporary electrophysiology standards and randomized trial data supporting uninterrupted or minimally interrupted anticoagulation strategies during left atrial catheter procedures [23]. Importantly, no anticoagulation-related procedural complications were observed, reinforcing the safety of maintaining therapeutic anticoagulation during transseptal access.

Our study has several limitations. First, it is a retrospective single-center analysis with a small sample size, which may limit the generalizability of our findings to larger and more diverse populations. The limited number of patients also precluded a robust subgroup analysis, such as comparisons based on gender, comorbidities, or differing clinical presentations. Additionally, the cohort primarily consisted of patients with bileaflet prosthetic valves, and the absence of other valve types restricted our ability to evaluate variability in Doppler–catheter relationships across different designs. Another limitation is the lack of simultaneous Doppler and catheter-based measurements, as these were performed on different days. This temporal separation could introduce variability due to hemodynamic changes such as fluctuations in heart rate, blood pressure, or fluid status.

Furthermore, the influence of sedation or anesthesia during catheterization on hemodynamic parameters was not systematically assessed, potentially impacting the observed gradients. Finally, while our study provides valuable insights into the discordance between echocardiographic and invasive measurements, the findings should be interpreted cautiously, given both techniques’ inherent technical and methodological limitations. Future studies with larger cohorts and standardized protocols for simultaneous measurements are needed to validate and expand upon these results.

In conclusion, our findings confirm that echocardiography-derived TVG is often higher than catheter-based measurements in patients with aortic valve prostheses. Multiple factors influence this discrepancy. While echocardiography remains an essential tool for the noninvasive evaluation of prosthetic valve function, invasive catheterization should be considered in cases where Doppler findings are inconclusive or when precise hemodynamic data are needed to guide clinical decisions. A comprehensive multimodal approach is critical to optimizing patient outcomes and ensuring accurate diagnosis.

## 5. Conclusions

Echocardiography remains the cornerstone of prosthetic aortic valve assessment; however, it frequently overestimates transvalvular gradients when compared with invasive measurements. In this cohort, Doppler-derived peak gradients were significantly higher than invasive peak-to-peak gradients. The effective orifice area index (EOAI) <0.50 cm^2^/m^2^ emerged as a robust marker of severe hemodynamic obstruction, confirmed invasively. Although limited by small sample size and inclusion of bioprosthetic cases, subgroup analysis demonstrated consistent trends across valve types.

**Central Message:** Echocardiography significantly overestimated transvalvular gradients compared to invasive catheterization. EOAI < 0.50 cm^2^/m^2^ predicted severe hemodynamic obstruction.

**Perspective Statement:** This study demonstrates that echocardiographic evaluation tends to overestimate transvalvular gradients in patients with surgical AVR. It highlights the diagnostic utility of EOAI < 0.50 cm^2^/m^2^ as a marker of true hemodynamic obstruction, emphasizing the value of invasive transseptal catheterization for confirmation in patients with discordant imaging findings.

**Abbreviated Legend for Central Picture:** Echo overestimates TVG; EOAI < 0.50 cm^2^/m^2^ predicts severe catheter-based gradient.

## Figures and Tables

**Table 1:** Demographic, clinical, and laboratory characteristics of the study population.

Variables	(*n* = 14)
Age, years	49.2 ± 16
Gender, female	10 (71.4%)
Body weight, kg	80 ± 9.0
Height, m	165.92 ± 7.9
BMI, kg/m^2^	29.3 ± 5.3
BSA, m^2^	1.91 ± 0.10
Coronary artery disease	2 (14.3%)
Stroke	1 (7.1%)
Hypertension	4 (28.6%)
Index AVR indication	1 (7.1%) 13 (92.9%)
Aortic stenosis + regurgitation	
Aortic stenosis	
Prosthetic valve type	
Mechanic prosthesis	12 (85.7%)
Bioprothesis	2 (14.3%)
Prosthetic valve size	
19 mm	3 (21.4%)
21 mm	11 (78.6%)
Previous cardiovascular surgery	1.3 ± 0.6
Echocardiographic parameters	
Ascending aorta diameter, mm	33.4 ± 3.43
LVEDD, mm	46.14 ± 3.39
LVEF, %	61.57 ± 4.53%
Aortic valvular gradient, peak, mmHg	77 ± 13.1
Aortic valvular gradient, mean, mmHg	44.2 ± 8.9
AVA	0.87 (0.62–1.23)
EOA index	0.47 (0.34–0.62)
Time interval between AVR and cardiac catheterization, years	6.7 (2.5–11.5)
Time interval between echocardiography and cardiac catheterization, days	7.2 (2–19)
NYHA class	
I	2 (14.3%)
II	12 (85.7%)
BNP level	89.80 ± 67.5
Invasive transaortic peak-to-peak gradient, mmHg	47.5 ± 21.74

Data shown mean ± SD, median (min-max) and count (percentage). AVA: Aortic valve area; AVR: Aortic valve replacement; BNP: Brain natriuretic peptide; DVI: Doppler velocity index; EOA: effective orifice area; LVEDD: Left ventricular end diastolic diameter; LVEF: Left ventricular ejection fraction; NYHA: New York Heart Association.

**Table 2:** Transvalvular Gradient Measurements: Echocardiography vs. Invasive Catheterization.

Patient	Age/Gender (Years)	Echo Mean Gradient (mmHg)	Echo Peak Gradient (mmHg)	Invasive Peak to Peak Gradient (mmHg)	Doppler Velocity Index	Valve Type	Company Name/Size	Fluoroscopic Leaflet Motion
1	66/F	42	75	70	0.24	SAVR/Bileaflet	St. Jude/21	Normal
2	52/F	52	97	70	0.26	SAVR/Bileaflet	St. Jude/19	Reduced
3	25/F	41	70	65	0.23	Bioprosthetic valve	St. Jude/21	Normal
4	42/F	52	95	15	0.33	SAVR/Bileaflet	St. Jude/19	Normal
5	57/F	61	87	30	0.31	Bioprosthetic valve	St. Jude/21	Normal
6	67/F	47	84	32	0.35	SAVR/Bileaflet	St. Jude/21	Normal
7	40/M	42	77	35	0.29	SAVR/Bileaflet	Medtronic-Hall/21	Normal
8	57/F	42	66	40	0.30	SAVR/Bileaflet	St. Jude/21	Normal
9	26/M	32	56	60	0.35	SAVR/Bileaflet	St. Jude/21	Normal
10	24/M	35	68	54	0.37	SAVR/Bileaflet	St. Jude/21	Normal
11	75/F	60	95	80	0.27	SAVR/Bileaflet	St. Jude/19	Reduced
12	58/F	38	59	19	0.33	SAVR/Bileaflet	St. Jude/21	Normal
13	57/F	39	72	19	0.20	SAVR/Bileaflet	St. Jude/21	Normal
14	43/M	37	77	65	0.20	SAVR/Bileaflet	Medtronic-Hall/21	Normal

Abbreviations: SAVR: Surgical Aortic Valve Replacement.

## Data Availability

The data supporting this study’s findings are available on request from the corresponding author [U.C.].

## Supplementary Materials

The following supporting information can be downloaded at: https://www.mdpi.com/article/10.3390/diagnostics15232980/s1, Supplementary Video S1. Simultaneous aortic valve gradient measurement is performed using a pigtail catheter placed into the left ventricle via transseptal puncture and another pigtail catheter placed transaortically.
